
JOURNAL INFORMATION
==============================
NLM Title Abbreviation: Am J Trop Med Hyg
Journal ID: Am J Trop Med Hyg
NLM Journal ID: 0370507
Journal ID: tpmd

The American Journal of Tropical Medicine and Hygiene

ISSN: 0002-9637
EISSN: 1476-1645
Publisher: The American Society of Tropical Medicine and Hygiene

ARTICLE INFORMATION
==============================
PMCID: PMC10993790
DOI: 10.4269/ajtmh.1104stoc
Article ID: tpmd1104stoc
Article version: 1
Subjects: Other

Contents

Electronic publication date: 2024 Apr 2
Print publication date: 2024 Apr
Volume: 110
Issue: 4 Suppl
Issue title: Interventions for the Elimination and Prevention of Re-Establishment of Malaria
First page: ii
Last page: ii
Copyright: © 2024, The American Society of Tropical Medicine and Hygiene
Copyright year: 2024
License: This is an open-access article distributed under the terms of the Creative Commons Attribution (CC-BY) License, which permits unrestricted use, distribution, and reproduction in any medium, provided the original author and source are credited.
License URL: https://creativecommons.org/licenses/by/4.0/

Page count: 1

==============================

### Interventions for the Elimination and Prevention of Re-Establishment of Malaria

### Guest Editor: Kammerle Schneider

### CONTENTS

**EDITORIAL**

**Systematic Reviews and Meta-Analyses to Inform Recommendations for the Final Phase of Elimination and Prevention of Re-Establishment of Malaria**
N. Regina Rabinovich and Richard W. Steketee 1

**ARTICLES**

**Development of WHO Recommendations for the Final Phase of Elimination and Prevention of Re-Establishment of Malaria**
Kevin Marsh, Elie Akl, Jane Achan, Mohammed Alzahrani, J. Kevin Baird, Teun Bousema, Dionicia Gamboa, Marcus Lacerda, Kamini Mendis, Melissa Penny, Allan Schapira, Siv Sovannaroth, Chansuda Wongsrichanalai, Amanda Tiffany, Xiaohong Li, Erin Shutes, David Schellenberg, Pedro Alonso, The WHO Malaria Elimination Steering Group, and Kim A. Lindblade 3

**Development of Systematic Reviews to Inform WHO's Recommendations for Elimination and Prevention of Re-Establishment of Malaria: Methodology**
Maria Tusell, Laura C. Steinhardt, Julie Gutman, Zachary D. Schneider, Beena Bhamani, Monica P. Shah, Elisabet Martí Coma-Cros, John E. Gimnig, Koya C. Allen, Elie A. Akl, and Kim A. Lindblade 11

**Mass Drug Administration to Reduce Malaria Transmission: A Systematic Review and Meta-Analysis**
Zachary D. Schneider, Monica P. Shah, Marisa C. Boily, Alexandra L. Busbee, Jimee Hwang, Kim A. Lindblade and Julie R. Gutman 17

**Mass Drug Administration: Contextual Factor Considerations**
Zachary D. Schneider, Alexandra L. Busbee, Marisa C. Boily, Monica P. Shah, Jimee Hwang, Kim A. Lindblade and Julie R. Gutman 30

**Mass Relapse Prevention to Reduce Transmission of *Plasmodium vivax*—A Systematic Review**
Monica P. Shah, Nelli Westercamp, Kim A. Lindblade and Jimee Hwang 38

**Mass Testing and Treatment to Accelerate Malaria Elimination: A Systematic Review and Meta-Analysis**
Beena Bhamani, Elisabet Martí Coma-Cros, Maria Tusell, Vita Mithi, Elisa Serra-Casas, Nana Aba Williams, Kim A. Lindblade, and Koya C. Allen 44

**Targeted Testing and Treatment To Reduce Human Malaria Transmission in High-Risk Populations: A Systematic Review**
Beena Bhamani, Elisabet Martí Coma-Cros, Maria Tusell, Vita Mithi, Elisa Serra-Casas, Nana Aba Williams, Kim A. Lindblade, and Koya C. Allen 54

**Targeted Drug Administration to Reduce Malaria Transmission: A Systematic Review and Meta-Analysis**
Maria Tusell, Elisabet Martí Coma-Cros, Beena Bhamani, Vita Mithi, Elisa Serra-Casas, Nana Aba Williams, Kim A. Lindblade, and Koya C. Allen 65

**Targeted Test and Treat at Point of Entry to Reduce Importation of Malaria Parasites: A Systematic Review**
Elisabet Martí Coma-Cros, Maria Tusell, Beena Bhamani, Vita Mithi, Elisa Serra-Casas, Nana Aba Williams, Kim A. Lindblade, and Koya C. Allen 73

**Reactive Case Detection and Treatment and Reactive Drug Administration for Reducing Malaria Transmission: A Systematic Review and Meta-Analysis**
Laura C. Steinhardt, Achyut KC, Amanda Tiffany, Elizabeth M. Quincer, Leah Loerinc, Nicolas Laramee, Amy Large, and Kim A. Lindblade 82

**Reducing Malaria Transmission through Reactive Indoor Residual Spraying: A Systematic Review**
John E. Gimnig, Laura C. Steinhardt, Taiwo Samson Awolola, Daniel Impoinvil, Sarah Zohdy, and Kim A. Lindblade 94

For more information about
the American Society of Tropical Medicine and Hygiene
please visit us at www.astmh.org

The American Journal of Tropical Medicine and Hygiene is
online at www.ajtmh.org
